# Efficacy and Safety of Bruton Tyrosine Kinase Inhibitor Monotherapy Compared with Combination Therapy for Chronic Lymphocytic Leukemia and Small Lymphocytic Lymphoma: A Systematic Review and Meta-Analysis

**DOI:** 10.3390/cancers15071996

**Published:** 2023-03-27

**Authors:** Thi Thuy Nguyen, Nguyen Thanh Nhu, Van Khoi Tran, Tran Thuc Huan Nguyen, Chiou-Feng Lin

**Affiliations:** 1 International Ph.D. Program in Medicine, College of Medicine, Taipei Medical University, Taipei 110, Taiwan; ntthuy.ub@hueuni.edu.vn (T.T.N.);; 2 Department of Oncology, Hue University of Medicine and Pharmacy, Hue University, Hue 49120, Vietnam; 3 Faculty of Medicine, Can Tho University of Medicine and Pharmacy, Can Tho 94117, Vietnam; 4 Department of Surgery, Hue University of Medicine and Pharmacy, Hue University, Hue 49120, Vietnam; 5 Department of Microbiology and Immunology, School of Medicine, College of Medicine, Taipei Medical University, Taipei 110, Taiwan; 6 Graduate Institute of Medical Sciences, College of Medicine, Taipei Medical University, Taipei 110, Taiwan; 7 Core Laboratory of Immune Monitoring, Office of Research & Development, Taipei Medical University, Taipei 110, Taiwan

**Keywords:** chronic lymphocytic leukemia, small lymphocytic lymphoma, Bruton tyrosine kinase inhibitor, systematic review, meta-analysis

## Abstract

**Simple Summary:**

The approval of combination treatments such as chemoimmunotherapy was a breakthrough in managing chronic lymphocytic leukemia and small lymphocytic lymphoma (CLL/SLL) patients. However, benefits remain suboptimal. A Bruton tyrosine kinase inhibitor (BTKi) is an effective treatment for these patients. This meta-analysis reviewed published studies to compare the efficacy and safety of BTKis versus combination therapy in CLL/SLL. One thousand five hundred ten patients from four trials were analyzed. BTKi monotherapy was associated with significantly longer progression-free survival (PFS) and an improved overall response rate without excess toxicity. We observed similar benefits for PFS among patients with high-risk diseases. In addition, patients receiving second-generation BTKis (acalabrutinib or zanubrutinib) had fewer grade ≥ 3 adverse events than those receiving the combination treatment. Further studies are essential to enhance these results and determine the optimal therapy for managing CLL/SLL patients. This study may help hematologists plan the treatment of CLL/SLL.

**Abstract:**

The effectiveness and safety of combination treatments such as chemoimmunotherapies in chronic lymphocytic leukemia and small lymphocytic lymphoma (CLL/SLL) remain controversial. Bruton tyrosine kinase inhibitors (BTKis) are an effective therapy for CLL/SLL patients. This meta-analysis aimed to compare the efficacy and safety of BTKis versus combination therapy in CLL/SLL patients. We searched the PubMed, Cochrane, Medline, and Embase databases through February 2023 for relevant randomized controlled trials (RCTs). Four RCTs (including 1510 patients) were found and met the inclusion criteria. Progression-free survival (PFS) was significantly improved with BTKis when compared to the combination therapy (hazard ratio (HR), 0.30; 95% confidence interval (CI), 0.22–0.40), while a pooled analysis of overall survival did not favor single-agent BTKis over the combination therapy (HR, 0.87; 95% CI, 0.67–1.15). We observed consistent benefits for PFS among patients with high-risk disease characteristics. Although there was no difference in complete response between the two arms (risk ratio (RR), 0.54; 95% CI, 0.20–1.46), BTKi use was related to a better overall response rate (RR, 1.10; 95% CI, 1.04–1.16). The risk of grade ≥3 adverse events (AEs) was comparable between the two arms (RR, 0.82; 95% CI, 0.55–1.23). However, the risk of grade ≥3 AEs was significantly lower in the second-generation BTKi group than in the combination therapy group (RR, 0.73; 95% CI, 0.54–0.98). Overall, BTKis have superior efficacy compared to the combination regimens in patients with untreated or treated CLL/SLL without excess toxicity. Further studies are needed to confirm these results and determine the optimal therapy for managing patients with CLL/SLL.

## 1. Introduction

Chronic lymphocytic leukemia (CLL) and its counterpart, small lymphocytic lymphoma (SLL), are indolent B-cell lymphomas traditionally treated with combination regimens, such as chemoimmunotherapy (CIT), as the standard treatment [1,2]. Combination therapies of the investigator’s choice, such as venetoclax–rituximab, venetoclax–obinutuzumab, bendamustine–rituximab (BR), chlorambucil–obinutuzumab (CLBO), and idelalisib–rituximab (IR), have been used as therapeutic options for previously untreated or relapsed/refractory CLL [3,4,5,6,7,8]. The BR regimen is considered an appropriate second-line therapy for CLL patients in many countries [9], and IR has been used as a choice for patients who are intolerant to ibrutinib [10,11]. Recently, the Food and Drug Administration (FDA) approved venetoclax–obinutuzumab as a first-line therapy for comorbid CLL patients, whereas venetoclax–rituximab treatment for 24 months is recommended as one of the most common treatment options for relapsed/refractory disease. The combination of BR was associated with a median progression-free survival (PFS) of 11–18 months [4,7]. Treatment with CLBO improved outcomes in patients with untreated CLL and coexisting conditions (a median PFS of 26.7 months) [6]. Studies using IR reported a PFS of 20.3 months but featured treatment-limiting adverse events (AEs) [5,12]. CIT is correlated with undesirable events in many patients, and the risk of toxicities increases with age [13]. Recently, therapy for CLL or SLL patients has appeared to shift from CIT or anti-CD20-based combination therapy to targeted oral monotherapy with small-molecule inhibitors [14]. These effective targeted oral therapies have acceptable toxic effects and could be of value in patients with CLL and SLL.

Novel immunotherapeutics with B-cell receptor signaling inhibitors used against Bruton tyrosine kinase (BTK), such as ibrutinib, acalabrutinib, and zanubrutinib, are rapidly being included in the clinical paradigm of patients with either untreated or relapsed/refractory CLL and SLL [15,16,17,18]. Ibrutinib is a first-in-class selective and irreversible BTK inhibitor (BTKi) that inhibits CLL-associated cell signaling, adhesion, propagation, and homing in vitro and in vivo [19,20,21]. Treatment with ibrutinib monotherapy led to a median PFS of 52 months among those who had relapsed/refractory CLL [22]. Therefore, ibrutinib received FDA approval in 2013 as a treatment option for CLL or SLL patients [15,23]. Ibrutinib-based treatment with or without anti-CD20 antibodies improved outcomes compared to CIT regimens, including PFS [24,25] and overall survival [24]. However, cardiovascular toxicity and a risk of bleeding are the main concerns with continuous ibrutinib use [26,27]. Since the approval of first-generation BTKis, other targeted BTKis have shown significantly improved endpoints in large randomized trials and have been approved for the management of CLL and SLL. Acalabrutinib is a second-generation covalent BTKi with an increased selectivity over family kinases [28] which was approved in the US for any line of treatment of adults with CLL or SLL in 2019 [29]. Acalabrutinib, with or without the anti-CD20 antibody Obinutuzumab, showed superior outcomes in comparison to CIT treatment, demonstrating a similar risk of bleeding complications and reduced cardiovascular toxicity compared with ibrutinib [30]. Zanubrutinib is a novel selective covalent inhibitor of BTK with limited activity against alternative targets versus ibrutinib [31], and it demonstrated activity in early phase clinical trials in B-cell lymphomas including CLL and SLL. Hence, zanubrutinib was approved by the FDA for managing adults with mantle cell lymphoma in 2019; marginal zone lymphoma and Waldenström macroglobulinemia in 2021; and CLL and SLL in 2023 [32,33]. Pivotal phase III RCTs showed that zanubrutinib had superior outcomes and lower atrial fibrillation rates than ibrutinib in patients with relapsed/refractory CLL [34].

The benefit and safety of BTKi monotherapy relative to standard combination treatments remain a critical consideration. Combination therapy has been considered a backbone in the treatment of CLL or SLL. Additionally, no published systematic review or meta-analysis has compared the benefit and safety of BTKi monotherapy versus combination therapy in CLL and SLL patients, including patients with high-risk disease characteristics. Hence, this meta-analysis aimed to summarize and compare the clinical efficacy and safety of BTKi alone treatment versus the combination treatment in patients with CLL and SLL.

## 2. Materials and Methods

### 2.1. Registration and Protocol

The systematic review protocol was registered on the “International Prospective Register of Systematic Review” under CRD42023396006. Moreover, the review was conducted and written as required by the “Preferred Reporting Items for Systematic Reviews and Meta-Analyses” (PRISMA) 2020 guidelines [35].

### 2.2. Data Sources and Search Strategies

The PubMed, Cochrane Library, Embase, and MEDLINE databases were searched with the terminological formula: “chronic lymphocytic leukemia” or “small lymphocytic lymphoma” AND “bruton tyrosine kinase inhibitor” or “BKT” AND “randomized controlled trial”. We searched through February 2023 to retrieve relevant publications without restrictions on type, language, or country (a detailed search strategy is demonstrated in Table S1). We also scanned the reference lists of the eligible studies to search for other relevant records. Two authors performed the search independently. Any disagreements were resolved via discussion with another author.

### 2.3. Study Selection

We checked all randomized controlled trials (RCTs) that compared BTKi monotherapy (e.g., ibrutinib, acalabrutinib, and zanubrutinib) with combination therapy for treatment-naïve and/or relapsed/refractory CLL/SLL. In this setting, the combination treatment could have included BR, CLBO, IR, venetoclax–rituximab, and venetoclax–obinutuzumab for CLL/SLL [36]. All RCTs were selected without limitations on the country, study quality, or median follow-up time. To obtain a homogeneous population and avoid potential bias, we did not include conference abstracts, reviews, notes, letters, case reports, comments, or cell/animal studies. Furthermore, trials that did not report complete data, trials with small sample sizes, ongoing studies, studies without head-to-head comparisons, and studies with adjusted comparisons using patient-level data from two sources were excluded. Titles and abstracts were reviewed to remove duplicate and irrelevant records lacking the requisite information on Endnote X9 (Clarivate, Philadelphia, PA, USA). The full text of each trial was read, and those that met the eligibility criteria were included.

### 2.4. Data Extraction Process and Risk of Bias Assessment

Two independent evaluators conducted the data extraction procedure. First, the included publications’ full texts, figures, tables, and supplementary data (Tables S1–S5 and Figures S1–S5) were read to extract information, including clinical features and outcomes. If data were unavailable, we contacted the corresponding authors. Any disagreements between the two evaluators were resolved via discussion with a third evaluator. For data extraction, the following data related to RCTs and patient characteristics were collected: first author, year of publication, trial design, accrual enrolled period, ClinicalTrials.gov identifier, response and safety assessment, randomization and masking, procedures, sample size, time of initial response assessment, median follow-up time, patient characteristics (sex; Eastern Cooperative Oncology Group performance status (ECOG PS); Rai or Binet stage; immunoglobulin heavy chain gene (IGHV) status; and high-risk genomic features, including 11q deletion (del[11q]), 17p deletion (del[17p]), tumor protein 53 (TP53) mutation, and complex karyotype), primary outcomes, and secondary and additional secondary outcomes.

The “Cochrane Handbook for Systematic Reviews of Interventions (version 6.3)” was used to check the methodological quality and the risk of bias of the eligible trials [37]. Two authors independently assessed, cross-checked, and filled out the available datasheets for the Cochrane risk-of-bias tool for randomized trials (ROB 2) checklists. The randomization process, deviations from intended interventions, missing outcome data, measurement of the outcome, and selection of the reported results were examined carefully. Finally, disagreements were resolved via discussion with another evaluator.

### 2.5. Study Outcome Evaluation

The primary outcomes of interest were PFS and overall survival (OS), evaluated by an independent review committee (IRC) in the intention-to-treat analysis. PFS was defined as the time from the randomization date to progressive disease or death from any cause, and OS was calculated as the date from the random assignment until death due to any cause. Key secondary outcomes of interest included the IRC-assessed overall response rate (ORR), complete response (CR), time to next treatment, and toxicity. The ORR was defined as the proportion of patients with CR, CR with incomplete hematological recovery, nodular partial response (PR), PR with lymphocytosis, or PR. The CR rate included CR and CR with incomplete hematological recovery. Response assessments by the IRC and/or investigators were conducted for CLL per the “International Workshop on Chronic Lymphocytic Leukemia” (IWCLL) 2008 criteria [38], and for SLL per the Lugano classification for lymphoma 2014 [39]. AEs were classified per “the National Cancer Institute Common Terminology Criteria for Adverse Events, version 4.03”. Time to next treatment was defined as the time from random assignment to the institution of non–protocol-specified treatment for CLL/SLL, the first dose of BTKi monotherapy for patients in the combination therapy group who crossed over, or death).

### 2.6. Statistical Analysis

The hazard ratios (HRs) for BTKi monotherapy versus combination therapy were calculated using R software to compare the efficacy endpoints (R Foundation for Statistical Computing; Vienna, Austria). The treatment effect (TE) and standard errors of TE (seTE) determined from 95% confidence intervals (CIs), estimating a normal distribution in a log-transformed test in the analysis of HR. An HR of <1.0 was in favor of BTKi monotherapy. If HRs were not reported in the full text or supplementary information but the corresponding Kaplan–Meier curves were reported, we used the algorithm described by Tierney et al. [40] to calculate the HR from digitized curves in combination with the patients at risk and the sum of events. Risk ratios (RRs) and their 95% CIs for binary endpoints were estimated using the standard Mantel–Haenszel method. A random effects meta-analysis was used to pool the effect sizes of each endpoint due to heterogeneity between studies. Hedges’s Q and I^2^ statistics were calculated to estimate the magnitude of heterogeneity, with an I^2^ higher than 50% considered significant heterogeneity. Subgroup analyses were calculated to explore the sources of heterogeneity among trials.

## 3. Results

### 3.1. Literature Search and Study Selection

**Figure 1:**
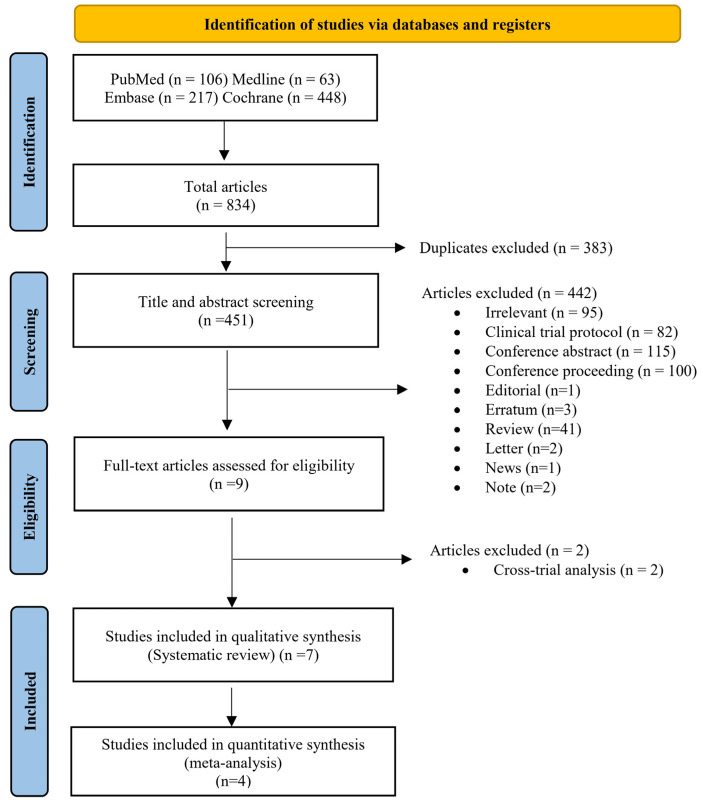
PRISMA flowchart summarizing the study selection process.

A total of 834 publications were collected through an electronic and manual search in the PubMed (n = 106), MEDLINE (n = 63), Embase (n = 217), and Cochrane Library (n = 448) databases. Then, 451 records were screened for their titles and abstracts. Of these records, 442 were excluded due to irrelevancy (n = 95) or because they were clinical protocols (n = 82), conference abstracts (n = 115), conference proceedings (n = 100), editorials (n = 1), errata (n = 3), reviews (n = 41), letters (n = 2), news (n = 1), or notes (n = 2). Nine articles were then determined to be potentially eligible. After eliminating two papers due to cross-trial analysis, seven publications were considered for data extraction. As three publications were determined to have long-term follow-ups published over time and with different endpoints, only four international RCTs with outcome analysis results were pooled in the final analysis (Figure 1).

### 3.2. Study Features

A total of four RCTs met the inclusion criteria: the ALLIANCE [25,41], ASCEND [42,43], ELEVATE-TN [30,44], and SEQUOIA [45] trials. All were published from 2018 to 2022 and were conducted in multicenter academic or community hospitals in multiple countries between 2013 and 2019. The characteristics of the four included trials are described in Table 1.

### 3.3. Description of Patients

Our meta-analysis included 1510 patients (40 SLL patients and 1470 CLL patients), with 1252 patients aged ≥65 years. Baseline demographic and condition features in each arm included a median age of 70 years (IQR 32–90), with males comprising 64%. Of the total 1510 patients, 1396 (92%) had an ECOG PS score of 0 or 1, 632 (42%) of 1510 individuals had high-risk conditions according to their Rai or Binet stage, and 872 (64%) of 1373 patients with evaluable results had unmutated an IGHV. In addition, del[17p] was present in 106 (7%) patients, del[11q] was present in 304 (20%) individuals, a TP53 mutation was present in 172 (12%) patients, and 242 (24%) of 994 patients had complex karyotypes. Most studies examined older patients with CLL or SLL and coexisting conditions except for the ASCEND trial, which recruited relapsed/refractory patients with CLL. The median follow-up duration ranged from 26.2 to 46.9 months (Table 2).

### 3.4. BTK Inhibitor Monotherapy versus Combination Therapy

After assessing their eligibility, patients were randomly assigned in a 1:1 ratio to BTKi alone or combination treatment. All patients in the BTKi group received either a daily oral dose of 420 mg ibrutinib, 100 mg bid for acalabrutinib, or 320 mg daily for zanubrutinib in 28 day cycles until disease progression or unacceptable toxic effects. In the combination therapy arm, while patients in the ELEVATE-TN trials received six cycles of the CLBO regimen [30,44], most patients in the other three RCTs were administered six cycles of the BR protocol. Notably, idelalisib (150 mg) was administered orally twice daily until disease progression or unacceptable toxicity in 119 CLL patients in the ASCEND trials. The crossover was included in the protocol of all RCTs and permitted the combination therapy of patients demonstrating disease progression to receive BTKi monotherapy. All patients in the combination therapy arm who crossed over to receive BTKi monotherapy continued to be followed for OS in the combination therapy arm in all four studies.

### 3.5. Risk of Bias

All four trials were judged to have some concerns about bias because of deviations from the intended intervention (Table 3). Although these RCTs were open-label studies, responses and progression were evaluated generally by the IRC, which was blinded to treatment group assignments.

### 3.6. Primary Outcomes of Interest

**Figure 2:**
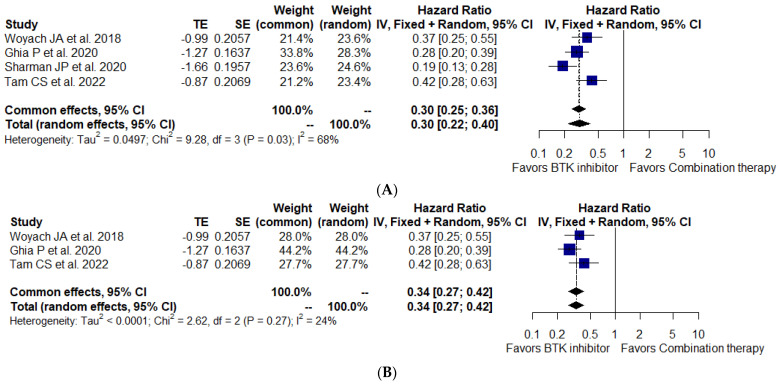
Forest plot for progression – free survival (**A**) and sensitivity analysis of progression – free survival (**B**) [25,30,42,45].

**Figure 3:**
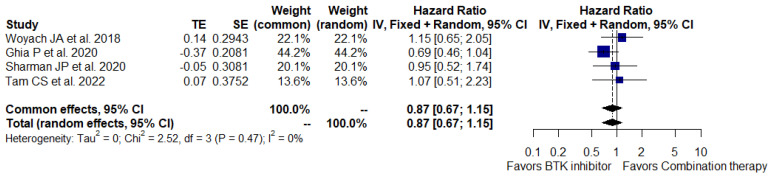
Forest plot for overall survival [25,30,42,45].

Data from all studies were available for the analysis of PFS and OS in 1470 CLL and 40 SLL patients. PFS was significantly improved with BTKi monotherapy compared to the combination therapy (HR, 0.30; 95% CI, 0.22–0.40; I^2^ = 68%; *p* = 0.03; 1510 patients, four studies) (Figure 2A). There was a high degree of heterogeneity in the results across these studies, and a sensitivity analysis using a non-CLBO regimen as a control group was conducted to detect potential sources of bias. The sensitivity analysis demonstrated a significant difference in PFS between the BTKi arm and the combination regimen arm (BR or IR) (HR, 0.34; 95% CI, 0.27–0.42; I^2^ = 24%; *p* = 0.27; 1154 patients, three studies) (Figure 2B). The advantage with respect to PFS with BTKi monotherapy remained consistent across the subgroup analysis among patients aged ≥ 65 years, male, Rai stage III/IV or Binet stage C, bulky disease ≥5 cm, unmutated IGHV, del[11q], and del[17p] (Table S2). However, the OS advantage was not statistically significant (HR, 0.87; 95% CI, 0.67–1.15; I^2^ = 0%; *p* = 0.47; 1510 patients, four studies) (Figure 3). Importantly, the time to next treatment was significantly improved with BTKi monotherapy when compared to combination therapy (HR, 0.31; 95% CI, 0.22–0.42; I^2^ = 0%; *p* = 0.51; 666 patients, two studies) (Figure S1).

### 3.7. Secondary Outcomes of Interest

**Figure 4:**
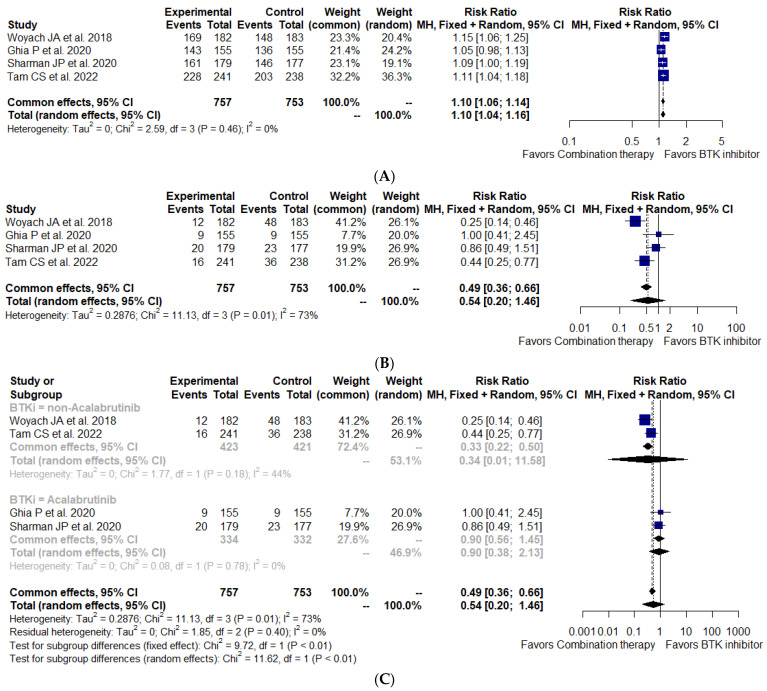
Pooled risk ratios for overall response (**A**), complete response (**B**), subgroup complete response (**C**) to treatment [25,30,42,45].

The treatment with BTKi alone was associated with a significantly better ORR (RR, 1.10; 95% CI, 1.04–1.16; I^2^ = 0%; *p* = 0.46; 1510 patients, four studies) (Figure 4A). However, the single-agent BTKi regimen did not improve the CR rate (RR, 0.54; 95% CI, 0.20–1.46; I^2^ = 73%; *p* = 0.01; 1510 patients, four studies) compared with the combination treatment (Figure 4B). Due to the substantial heterogeneity in this analysis, we performed a subgroup analysis between the acalabrutinib arm and the non-acalabrutinib arm. There was also no difference in the CR proportion between the two groups (Figure 4C).

The results showed no difference in the risk of AEs of any grade between the two groups (RR, 0.98; 95% CI, 0.91–1.06; I^2^ = 51%; *p* = 0.13; 1122 patients, three studies). All studies reported grade 3 or higher AEs (n = 1150). Similarly, the risk of grade ≥3 AEs was comparable between the two arms (RR, 0.82; 95% CI, 0.55–1.23; I^2^ = 92%; *p* <0.01; 1510 patients, four studies). However, there was a high level of heterogeneity in this analysis, and we conducted a subgroup analysis between first-generation and next-generation BTKis. The risk of grade ≥3 AEs was significantly lower in the second-generation BTKi monotherapy group than in the combination therapy group (RR, 0.73; 95% CI, 0.54–0.98; I^2^ = 64%; *p* = 0.06; 1122 patients, three studies). Regarding hematological toxicity, there was a decreased risk of grade ≥3 neutropenia in patients receiving the single-agent BTKi treatment versus the combination treatment (RR, 0.32; 95% CI, 0.18–0.58; I^2^ = 70%; *p* = 0.06; 1510 patients, four studies). Due to high heterogeneity, the subgroup analysis also demonstrated that there was a lower possibility of grade 3 or worse neutropenia in the BTKi therapy group than in the combination therapy group (RR, 0.28; 95% CI, 0.14–0.55; I^2^ = 44%; *p* = 0.17; 1171 patients, three studies) when treatment-naïve CLL or SLL patients were included in this analysis. In addition, the risk of grade 3 or higher thrombocytopenia was significantly lower in the BTKi arm than in the combination therapy arm (RR, 0.37; 95% CI, 0.20–0.70; I^2^ = 0%; *p* = 0.48; 1510 patients, four studies). Concerning nonhematologic AEs, the risk of grade ≥3 sepsis was significantly decreased among BTKi monotherapy patients compared to the combination regimen arm (RR, 0.55; 95% CI, 0.32–0.93; I^2^ = 0%; *p* = 0.86; 1510 patients, four studies). However, there was a significantly higher rate of secondary primary malignancies in the monotherapy group than in the combination therapy group (RR, 2.09; 95% CI, 1.01–4.36; I^2^ = 0%; *p* = 0.53; 1510 patients, four studies). The risk of grade 3 or higher AEs was comparable between the two groups. The results demonstrated no difference in the risk of cardiac AEs of grade ≥3 between the two arms (atrial fibrillation, ventricular tachycardia, sudden death, or hypertension) (Table 4). Importantly, all-grade (23%) or grade ≥3 (4.1%) AEs associated with infusion-related reactions were more common with the combination therapy than with BTKi treatment alone (Table S3).

## 4. Discussion

To the best of our knowledge, this is the first systematic review and meta-analysis examining the efficacy and safety of BTK inhibitor monotherapy compared to combination therapy for CLL and SLL. Overall, we demonstrated that the single-agent BTKi treatment had statistically significant superior outcomes compared with the combination regimens in patients with untreated or treated CLL and SLL. In addition, BTKi monotherapy was associated with significantly longer PFS, a longer time to the next treatment, and an improved ORR without excess toxicity. Importantly, patients receiving second-generation BTKis (acalabrutinib or zanubrutinib) had fewer grade ≥3 AEs than those receiving the combination treatment.

Despite the high level of heterogeneity in the PFS analysis, BTKi treatment alone still resulted in a longer PFS than the combination treatment. The benefits from the BTKi regimen in terms of PFS were also consistent with the sensitivity analysis focusing on the BR or IR regimen as the combination treatment. In addition, subgroup analyses showed a consistent PFS advantage with BTKi monotherapy regardless of age, sex, Rai or Binet stage, bulky disease, and high-risk genomic features. With long-term follow-up, the results of this meta-analysis confirm and extend the results of the published data, showing that BTKis are efficacious in CLL patients with del[11q], del[17p], and/or TP53 mutations because these patients have a generally poor response to CIT [46,47,48]. The findings of recent trials elicit a change in the standard treatment of CLL or SLL from conventional CIT to targeted agents [49,50]. As not all patients worldwide may have equal access to innovative medicines, our meta-analysis further supports this shift in the treatment paradigm for CLL or SLL patients.

The primary goal of a novel therapy is to improve OS, and the final analysis in our meta-analysis did not have sufficient power to assess a significant difference in OS between the two groups. However, the median OS was not met in any intervention arm in any included RCT. The results of our study suggest several possible factors. First, more effective therapies may contribute more to the survival prospects of younger individuals with CLL who have fewer comorbidities and are less likely to die from unrelated causes than older patients. Our studies examined elderly and frail populations, and the most common causes of death related to BTKi-containing regimens were unwitnessed or unexplained death and secondary malignancies other than CLL or SLL at the time of the final analysis [24,50]. In addition, given the short duration of the included studies and the low number of events, extended follow-up will be necessary to detect any difference in OS. Future investigations may be confounded by a crossover design.

A higher ORR was demonstrated for patients in the BTKi group versus those in the combination treatment group. Consistent with the IRC-assessed results, the investigator-assessed ORR was also significantly higher in the BTKi monotherapy arm than in the combination therapy arm [12,42,43,45]. However, the rate of CR was comparable between the two groups. The CR proportion could be underestimated in both arms because of the lack of the necessary bone marrow examination to verify the negative minimal residual disease in two RCTs [43,45].

As BTKis are administered until disease progression, their tolerance and long-term safety are critical, especially in CLL or SLL, which primarily affect elderly individuals with comorbidities. The current study detected comparable safety across the two groups, and no additional AEs were identified. These safety findings are similar to those of earlier studies in which most AEs in both treatment groups occurred within the first 6 months of the administration and became less common afterwards [27,51,52]. However, grade 3 and worse AEs occurred more often in patients treated with the combination regimens versus second-generation BTKis. Ibrutinib-related AEs can be therapeutically limiting [53,54] and are thought to be related to non-BTK target binding [17,55]. The tolerability profile of acalabrutinib or zanubrutinib implies that the lower off-target kinase activity in preclinical studies might transfer to clinical use [17,31,56]. A phase III randomized trial comparing acalabrutinib to ibrutinib in relapsed/refractory patients with CLL demonstrated again that acalabrutinib is better tolerated than ibrutinib [57]. The rates of grade 3 or higher neutropenia and thrombocytopenia were higher in the combination therapy group because hematological AEs occurred more commonly during treatment with the dual combination therapy [58].

Similarly, a decreased rate of sepsis was shown with BTKi versus combination treatment in this current analysis. Compared to the combination therapy, the rate of SPMs was higher with BTKi monotherapy; however, half of the nonmelanoma skin cancers were associated with BTKi treatment. The significant prevalence of SPMs reported with single-agent BTKis here is similar to earlier observations in BTKi treatment patients [59,60]. Moreover, SPMs are more common in CLL patients than other B-cell lymphoma patients [61]. The cardiovascular toxicity rate can be a notable limiting feature of BTKi administration and might be related to substantial cost, morbidity, and mortality [62,63]. In contrast to our results, previous trials reported that grade 3 or worse hypertension and atrial fibrillation events frequently occurred in patients undergoing CIT. In the ALLIANCE trial, a study comparing ibrutinib with BR, the risk of atrial fibrillation was 126% with ibrutinib and 3% with BR at the 24 month follow-up. However, a decreased rate of ventricular arrhythmias was demonstrated with acalabrutinib and zanubrutinib versus a first-generation BTKi or combination therapy in B-cell lymphomas [34,64]. These findings support the concept that second-generation BTKis’ lower suppression of off-target kinases may help to mitigate the higher risk of cardiac arrhythmias seen with ibrutinib [27,31,54].

The large sample size and sample pooling results are the key strengths of this meta-analysis. While three studies reported only an improved ORR and the fourth showed no advantage compared to combination therapy, a pooled analysis of all four RCTs demonstrated that BTKi monotherapy provides a statistically significant response advantage. Our study is the first systematic review and meta-analysis to show an improvement in efficacy and similar safety with BTKis versus combination therapy, which could soon affect treatment options for CLL. Another advantage of this study is that the findings may be applied to the elderly population, who are not candidates for transplantation. Patients aged >70 years are typically ineligible for high-dose chemotherapy and allogeneic stem cell transplantation due to significant comorbidities [65,66], or the treatments are not practical because of the high cost of chimeric antigen receptor T-cell therapy due to qualitative T-cell defects in patients with CLL [67,68]. Most of the examined patients in the four studies were older patients or those with coexisting conditions or relapsed/refractory. A subgroup analysis of PFS per age group demonstrated similar efficacy among patients aged >65 years. These findings can be applied to older patients and encourage trials on different targeted therapies in these populations. Importantly, given that all included trials were global, phase III, randomized controlled designs with blinded efficacy assessment by IRC and multicenter trials with extended follow-up durations, they were high-quality studies with a minimal risk of bias. As a result, we were able to achieve reliable conclusions.

Several limitations of our analysis should be considered. The key drawback is that BTKis are novel targeted agents that have been studied in recent randomized clinical trials only in the last 5 years, with results published only recently. As a result, only a few RCTs were included in this meta-analysis. Another considerable limitation is the variability in the study designs, which resulted in high heterogeneity between the studies, particularly between the ELEVATE-TN study and the other three studies. Although there are numerous similarities between the studies, two critical points differed among the trials: the generation of BTKi or the different combination treatments in each study and the inclusion of treatment-naïve or relapsed/refractory CLL patients. The patients and investigators not being masked to treatment assignment cause considerable potential for selection bias. Our method of grouping various combination regimens might also be a limitation, as the response to each combination treatment might differ depending on the patient’s characteristics. We acknowledge that mixing new targeted therapies and chemoimmunotherapy as a group control is difficult. However, all combination therapy regimens are considered a standard of care in CLL/SLL treatment [36]. We simply aimed to compare the monotherapy and combination therapy in managing CLL. Notably, after a search of the literature and study selection, most of the patients in the combination therapy arm received chemoimmunotherapy (anti-CD20 antibody combined with an alkylating agent chemotherapy). There were only 119 patients who received idelalisib (an inhibitor of PI3Kδ kinase) plus rituximab. There were not any included patients who received venetoclax–rituximab or venetoclax–obinutuzumab. In addition, we could not access individual patient data with comprehensive baseline characteristics, inhibiting us from performing a multivariable analysis to detect potential confounders that could affect the outcomes.

Despite several limiting factors in this study, our meta-analysis remains the only one to incorporate data from four extensive international trials with 1510 treatment-naïve or relapsed/refractory patients with CLL. The findings could impact clinical practice and the design of future clinical trials. First, more well-designed RCTs will soon be required to identify any differences in overall survival between the two groups. Second, to overcome BTKi resistance and intolerability, a phase III study comparing novel in-class BTKi agents is ongoing [69]. Finally, this meta-analysis did not include venetoclax–rituximab and venetoclax–obinutuzumab regimens, which would have promoted a direct comparison with BTKi monotherapy and will be addressed in further RCTs comparing venetoclax-based therapy to BTKi monotherapy in untreated or previously treated CLL patients.

Recently, there have been two head-to-head trials of acalabrutinib or zanubrutinib versus ibrutinib in relapsed/refractory CLL. While acalabrutinib showed noninferior PFS with fewer cardiac adverse events, the PFS was significantly longer among patients who received zanubrutinib than those who received ibrutinib. The safety profile of zanubrutinib was better than that of ibrutinib in patients with previously treated CLL [57,70]. A subsequent meta-analysis will be conducted soon to compare and evaluate the efficacy and safety of different BTK inhibitors in CLL/SLL patients. Regarding the impact on therapy costs, although continuous therapies are practical, they have financial toxicity to patients and payers in public systems [71].

The paradigm shift from chemoimmunotherapy to novel targeted therapies in CLL affects the prognostic impact of the biological markers. Döhner et al. revealed the prognostic effect of specific cytogenetic abnormalities discovered by FISH 20 years ago [72]. The del[11q] is seen in approximately 20% of individuals who require front-line therapy and is linked with poor results [73]. Early studies revealed that anti-CD20 might overcome the poor prognosis associated with this deletion, indicating that this del[11q] could be regarded as a predictive indicator for an enhanced response to CIT [6,74]. The researchers proposed that del[11q] may be a prognostic biomarker for improved efficacy in patients treated with ibrutinib. In any case, this discovery requires additional confirmation, and investigations with preclinical studies that include this change will aid in understanding del[11q]-related consequences on treatment response [48,75]. Despite the excellent outcomes produced by ibrutinib, some individuals do not react, and others relapse during therapy. A relevant mutation can be found in 70–80% of people with acquired resistance. These mutations happen in the ibrutinib–BTK binding site, generally at position C481S. Fewer common activating mutations in the PLCG2 pathway could be found [76,77]. Finally, acquired mutations may serve as indicators for ibrutinib resistance. The benefit of early diagnosis and the potential transition to alternative therapies has yet to be confirmed. Recent studies have revealed that the resistance mechanisms to acalabrutinib are comparable to those of ibrutinib, which is not surprising given that acalabrutinib binds to BTK at the exact location (C481S) [78]. From the patient’s perspective, it provides information that can aid in personal planning. Acquired BTK mutations are promising candidates for use as novel biomarkers for treatment failure.

## 5. Conclusions

This meta-analysis demonstrated that BTKi monotherapy has superior outcomes compared to the combination therapy and has a manageable safety profile in patients with untreated or relapsed/refractory CLL and SLL. These findings suggest using BTKis as an efficacious and well-tolerated treatment for patients with untreated or treated CLL or SLL (including individuals with high-risk disease characteristics) and suggest the potential use of other next-generation BTKis as new treatment options in the clinical setting.

## Figures and Tables

**Table 1:** Characteristics of the included randomized controlled trials.

	Alliance Woyach JA et al. 2018 [25]	Ascend Ghia P et al. 2020 [42]	Elevate-TN Sharman JP et al. 2020 [30]	Sequoia Tam CS et al. 2022 [45]
Study design	Phase III, MC, OL	Phase III, MC, OL	Phase III, MC, OL	Phase III, MC, OL
Enrolled period	December 2013–May 2016	February 2017–January 2018	September 2015–February 2017	October 2017–July 2019
ClinicalTrials.gov ID	NCT01886872	NCT02970318	NCT02475681	NCT03336333
Method of analysis	ITT	ITT	ITT	ITT
Response definition	iwCLL 2008 criteria	iwCLL 2008 criteria	iwCLL 2008 criteria	iwCLL 2008 criteria (CLL) or Lugano classification (SLL)
Safety assessment	NR	CTCAE v4.03	CTCAE v4.03	CTCAE v4.03
Inclusion criteria	Treatment-naive patients	Relapsed or refractory patients	Treatment-naive patients	Treatment-naive patients
Randomization	1:1	1:1	1:1	1:1
Masking	No	No	No	No
Randomization stratification	Risk factors for CLL	Presence or absence of del[17p] status, ECOG PS score, and lines of prior therapy received	Presence or absence of del[17p], ECOG PS score, and geographic region	Based on age, Binet stage, IGHV mutational status, and geographical region
Primary outcome	PFS	PFS	PFS	PFS
Secondary outcome	OS, ORR, CR, safety, etc	ORR, OS, DOR, safety, etc	ORR, OS, CR, safety, etc	ORR, OS, safety, etc
Definition PFS	The interval time from randomization until disease progression or death from any cause	The time from randomization until disease progression or death	The time from randomization until disease progression or death	The time from randomization until disease progression or death
Intervention arm	Ibrutinib 420 mg/day	Acalabrutinib 200 mg/day	Acalabrutinib 200 mg/day	Zanubrutinib 320 mg/day
Standard of care arm	Bendamustine 90 mg/m^2^ + Rituximab (375 mg/m^2^)	Bendamustine 70 mg/m^2^ or Idelalisib 300 mg/day + Rituximab (500 mg/m^2^)	Chlorambucil (0.5 mg/kg on day 1 and day 15) + Obinutuzumab (1000 mg)	Bendamustine 90 mg/m^2^ + Rituximab (500 mg/m^2^)
Sample size	365	310	356	479
Time of initial response assessment	NR	NR	Week 12	Week 12
Crossover design	Yes	Yes	Yes	Yes

Abbreviations: MC—multicenter; OL—open-label; ID—identifier; ITT—intention-to-treat; CLL—Chronic Lymphocytic Leukemia; SLL—small lymphocytic lymphoma; iwCLL—International Workshop on Chronic Lymphocytic Leukemia; CTCAE—the US National Cancer Institute Common Terminology Criteria for Adverse Events; ECOG PS—Eastern Cooperative Oncology Group performance status; IGHV—unmutated immunoglobulin heavy chain variable; PFS—progression-free survival; OS—overall survival; ORR—overall response rate; CR—complete response; DOR—duration of response; NR—not reported.

**Table 2:** Characteristics of patients included in the trials.

Study (First Author, Year)	BTK Inhibitor Group/Investigator’s Choice of Combination Therapy Group	Patient, n	Patient ≥ 65 years old	Patient age, median (range)	Sex, male, n (%)	ECOG PS (0–1), n (%)	ECOG PS (2), n (%)	High-Risk Disease (Rai/Binet Stage), n (%)	Treatment-Naïve Patients, n (%)	Relapsed or Refractory Patients, n (%)	Unmutated IGHV, n (%)	Del(17p), n (%)	Del(11q), n (%)	TP53 Mutation, n (%)	Complex Karyotype, n (%)	Follow-Up, Month, Median (Range)
Woyach JA et al. 2018 [25]	Ibrutinib Bendamustine + Rituximab	182 183	182 183	71 (65–89) 70 (65–86)	123 (68) 119 (65)	177 (97) 173 (95)	5 (3) 10 (5)	99 (54) 99 (54)	182 (100) 183 (100)	0 (0) 0 (0)	77/122 (63) 71/123 (58)	9/181 (5) 14/181 (8)	35/181 (19) 33/181 (18)	15/168 (9) 16/174 (9)	39/165 (24) 44/166 (27)	38 38
Ghia P et al. 2020 [42]	Acalabrutinib Bendamustine/Idelalisib +Rituximab	155 155	97 98	68 (32–89) 67 (34–90)	108 (70) 100 (65)	136 (88) 134 (86)	19 (12) 21 (14)	65 (42) 64 (41)	0 (0) 0 (0)	155 (100) 155 (100)	118/154 (77) 125/153 (82)	28/155 (18) 21/154 (14)	39/155 (25) 44/155 (29)	39/152 (26) 34/153 (22)	50/154 (32) 46/153 (30)	46.5 45.7
Sharman JP et al. 2020 [30]	Acalabrutinib Chlorambucil + Obinutuzumab	179 177	151 153	70 (66–75) 71 (67–76)	111 (62) 106 (59.9)	165 (92.2) 167 (94.4)	14 (7.8) 10 (5.6)	87 (48.6) 78 (44.1)	179 (100) 177 (100)	0 (0) 0 (0)	119 (66.5) 116 (65.5)	16 (8.9) 16 (9.0)	31 (17.3) 33 (18.6)	19 (10.6) 21 (11.9)	31 (17.3) 32 (18.1)	46.9 46.9
Tam CS et al. 2022 [45]	Zanubrutinib Bendamustine + Rituximab	241 238	196 192	70 (66–75) 70 (66–74)	154 (64) 144 (61)	226 (96) 218 (92)	15 (6) 20 (8)	70 (29) 70 (29)	241 (100) 238 (100)	0 (0) 0 (0)	125/234 (53) 121/231 (52)	2 (1) 0 (0)	43 (18) 46 (19)	15/232 (6) 13/223 (6)	NR	26.2 26.2

Abbreviations: BTK—Bruton tyrosine kinase; ECOG PS—Eastern Cooperative Oncology Group performance status; IGHV—unmutated immunoglobulin heavy chain variable; NR—not reported.

**Table 3:** Risk of bias evaluation of the included randomized trials using the ROB 2 tool.

Study	Bias arising from the Randomization Process	Bias due to Deviations from Intended Interventions	Bias due to Missing Outcome Data	Bias in the Measurement of the Outcome	Bias in the Selection of the Reported Result	The Overall Risk of Bias
Woyach JA et al. 2018 [25]	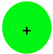	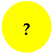	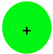	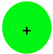	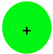	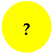
Ghia P et al. 2020 [42]	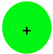	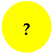	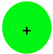	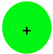	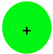	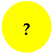
Sharman JP et al. 2020 [30]	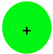	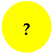	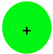	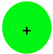	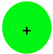	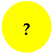
Tam CS et al. 2022 [45]	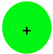	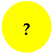	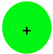	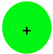	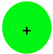	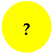

Abbreviation: 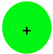
—Low risk; 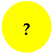
—Some concerns; 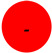
—High risk.

**Table 4:** Summary of pooled relative risk for grade 3 or higher adverse events.

Adverse Events	BTK Inhibitor		Combination Therapy		Risk Ratio	95% Confident Interval	I^2^ (%)	*p* -Value
	Events	Total	Event	Total				
AE any grade	543	573	530	549	0.98	0.91–1.06	51	0.13
AE of grade 3 or higher	452	753	535	725	0.82	0.55–1.23	92	<0.01
AE of grade 3 or higher subgroup	319	573	424	549	0.73	0.54–0.98	64	0.06
Anemia	54	753	49	725	1.06	0.45–2.49	30	0.23
Arthralgia	7	753	3	725	1.79	0.58–5.57	0	0.81
Diarrhea	11	753	42	725	1.58	0.22–11.56	43	0.15
Fatigue	16	753	12	725	1.29	0.92–1.82	0	0.97
Hemorrhage	21	753	8	752	2.01	0.54–7.44	0	0.40
Infection	150	753	127	725	1.18	0.68–2.03	52	0.10
Neutropenia	103	753	315	725	0.32	0.18–0.58	70	0.02
Neutropenia subgroup	74	599	257	572	0.28	0.14–0.55	44	0.17
Pneumonia	23	573	26	549	0.85	0.18–4.05	30	0.24
Sepsis	14	753	25	725	0.55	0.32–0.93	0	0.86
SPM	45	753	20	725	2.09	1.01–4.36	0	0.53
Thrombocytopenia	27	753	73	725	0.37	0.20–0.70	0	0.48
URTI	17	753	22	725	0.77	0.51–1.16	0	0.91
UTI	14	753	13	725	1.03	0.21–4.95	22	0.28
Cardiac adverse events	35	573	23	549	1.50	0.17–13.49	62	0.07
Atrial fibrillation	22	753	10	725	1.74	0.30–9.96	32	0.22
Ventricular tachycardia	2	753	0	725	-	-	-	-
Sudden death	7	420	2	403	-	-	-	-
Hypertension	80	753	43	725	1.64	0.60–4.51	36	0.19

Abbreviations. AE—adverse event; BTK—Bruton tyrosine kinase; SPM—secondary primary malignancies; URTI—upper respiratory tract infection; UTI—urinary tract infection.

## Data Availability

Data were extracted and analyzed from published articles available and accessible in the shared database. All datasets generated during the study are available upon reasonable request from the corresponding authors. The study protocol has been published (PROSPERO ID: CRD42023396006; www.crd.york.ac.uk/PROSPERO/ accessed on 1 February 2023) and is universally available.

## Supplementary Materials

The following supporting information can be downloaded at: https://www.mdpi.com/article/10.3390/cancers15071996/s1, Table S1: Search strategy (all fields included); Table S2: Summary of subgroup analysis of progression-free survival; Table S3: Summary of adverse events associated with infusion-related reactions; Table S4: Summary of adverse events associated with secondary primary malignancies; Table S5: PRISMA 2020 checklist; Figure S1: Forest plot for time to next treatment [30,42]; Figure S2: Forest plot for progression-free survival (subgroup different generation BTK inhibitors) [25,30,42,45]; Figure S3:Forest plot for overall survival (subgroup different generation BTK inhibitors) [25,30,42,45]; Figure S4: Pooled risk ratios for overall response (A) and complete response (B) (subgroup different generation BTK inhibitors) [25,30,42,45]; Figure S5: Pooled risk ratios for grade ≥3 adverse events [25,30,42,45].
