# Supplementary material for: Efficacy and Safety of Bruton Tyrosine Kinase Inhibitor Monotherapy Compared with Combination Therapy for Chronic Lymphocytic Leukemia and Small Lymphocytic Lymphoma: A Systematic Review and Meta-Analysis
Source: Cancers (Basel). 2023 Mar 27;15(7):1996. doi: 10.3390/cancers15071996 (PMC10093473; doi:10.3390/cancers15071996)
Supplement: Supplementary file 1 [file cancers-15-01996-s001.zip › cancers-2269337-supplementary.pdf]

Table S1. Search strategy (all fields included).

PubMed		
**No**	**Query**	**Results**
#1	"chronic lymphocytic leukaemia"[All Fields] OR "leukemia, lymphocytic, chronic, b cell"[MeSH Terms] OR ("leukemia"[All Fields] AND "lymphocytic"[All Fields] AND "chronic"[All Fields] AND "b cell"[All Fields]) OR "b-cell chronic lymphocytic leukemia"[All Fields] OR ("chronic"[All Fields] AND "lymphocytic"[All Fields] AND "leukemia"[All Fields]) OR "chronic lymphocytic leukemia"[All Fields] OR "CLL"[All Fields] OR ("leukemia, lymphocytic, chronic, b cell"[MeSH Terms] OR ("leukemia"[All Fields] AND "lymphocytic"[All Fields] AND "chronic"[All Fields] AND "b cell"[All Fields]) OR "b-cell chronic lymphocytic leukemia"[All Fields] OR ("small"[All Fields] AND "lymphocytic"[All Fields] AND "lymphoma"[All Fields]) OR "small lymphocytic lymphoma"[All Fields]) OR ("sign lang linguist"[Journal] OR "sll"[All Fields])	34,906
#2	"ibrutinib"[Supplementary Concept] OR "ibrutinib"[All Fields] OR "ibrutinib s"[All Fields] OR ("ibrutinib"[Supplementary Concept] OR "ibrutinib"[All Fields] OR "imbruvica"[All Fields] OR "ibrutinib s"[All Fields]) OR ("ibrutinib"[Supplementary Concept] OR "ibrutinib"[All Fields] OR "pci 32765"[All Fields]) OR "CRA-032765"[All Fields] OR ("acalabrutinib"[Supplementary Concept] OR "acalabrutinib"[All Fields] OR ("acalabrutinib"[Supplementary Concept] OR "acalabrutinib"[All Fields] OR "calquence"[All Fields]) OR ("acalabrutinib"[Supplementary Concept] OR "acalabrutinib"[All Fields] OR "acp 196"[All Fields])) OR ("zanubrutinib"[Supplementary Concept] OR "zanubrutinib"[All Fields] OR ("zanubrutinib"[Supplementary Concept] OR "zanubrutinib"[All Fields] OR "brukinsa"[All Fields]) OR ("zanubrutinib"[Supplementary Concept] OR "zanubrutinib"[All Fields] OR "bgb 3111"[All Fields])) OR ("tirabrutinib"[Supplementary Concept] OR "tirabrutinib"[All Fields] OR "Velexbru"[All Fields] OR ("tirabrutinib"[Supplementary Concept] OR "tirabrutinib"[All Fields] OR "ono 4059"[All Fields]) OR ("tirabrutinib"[Supplementary Concept] OR "tirabrutinib"[All Fields] OR "gs 4059"[All Fields])) OR "Orelabrutinib"[All Fields] OR ("pirtobrutinib"[Supplementary Concept] OR "pirtobrutinib"[All Fields] OR ("pirtobrutinib"[Supplementary Concept] OR "pirtobrutinib"[All Fields] OR "loxo 305"[All Fields])) OR ("Nemtabrutinib"[All Fields] OR ("arq531"[Supplementary Concept] OR "arq531"[All Fields] OR "arq 531"[All Fields]) OR "MK-1026"[All Fields]) OR ("agammaglobulinaemia tyrosine kinase"[MeSH Terms] OR ("agammaglobulinaemia"[All Fields] AND "tyrosine"[All Fields] AND "kinase"[All Fields]) OR "agammaglobulinaemia tyrosine kinase"[All Fields] OR ("bruton"[All Fields] AND "tyrosine"[All Fields] AND "kinase"[All Fields]) OR "bruton tyrosine kinase"[All Fields] OR "BTK"[All Fields])	7,018
#3	"randomized controlled trial"[Publication Type] OR "randomized controlled trials	780,435

	as topic"[MeSH Terms] OR "randomized controlled trial"[All Fields] OR "randomised controlled trial"[All Fields]	
#4	#1 AND #2 AND #3	106
**Embase**		
#1	'chronic lymphatic leukemia'/exp/mj	31,086
#2	'lymphocytic lymphoma'/mj	560
#3	#2 OR #3	31,275
#4	'bruton tyrosine kinase inhibitor'/exp/mj	9,111
#5	'ibrutinib'/exp/mj	3,870
#6	'acalabrutinib'/exp/mj	492
#7	'zanubrutinib'/exp/mj	267
#8	'tirabrutinib'/exp/mj	71
#9	'orelabrutinib'/exp/mj	31
#10	'pirtobrutinib'/exp/mj	50
#11	'nemtabrutinib'/exp/mj	10
#12	#4 OR #5 OR #6 OR #7 OR #8 OR #9 OR #10 OR #11	9,157
#19	'randomized controlled trial'/exp	749,487
#20	#3 AND #12 AND #19	217
**MEDLINE**		
#1	chronic lymphocytic leukemia.mp. or Leukemia, Lymphocytic, Chronic, B-Cell/ OR CLL.mp. OR small lymphocytic lymphoma.mp. OR SLL.mp.	29,362
#2	bruton tyrosine kinase inhibitor.mp. OR BTK.mp.	3,956
#3	ibrutinib.mp. OR Imbruvica.mp. OR PCI-32765.mp.	3,386
#4	acalabrutinib.mp. OR Calquence.mp. OR ACP-196.mp.	378
#5	zanubrutinib.mp. OR Brukinsa.mp. OR BGB-3111.mp.	221
#6	Tirabrutinib.mp. OR Velexbru.mp. OR ONO-4059.mp. OR GS-4059.mp.	90
#7	Orelabrutinib.mp.	16
#8	Pirtobrutinib.mp. OR LOXO-305.mp.	34
#9	Nemtabrutinib.mp. OR ARQ 531.mp.	13
#10	#2 OR #3 OR #4 OR #5 OR #6 OR #7 OR #8 OR #9	6,233
#21	randomized controlled trial.mp. or Randomized Controlled Trial/	626,019
#22	#1 AND #10 AND #21	63
**Cochrane library**		
#1	chronic lymphocytic leukemia OR CLL OR small lymphocytic lymphoma OR SLL	2,580
#2	Bruton tyrosine kinase inhibitor OR BTK	699
#3	Ibrutinib OR Imbruvica OR PCI-32765 OR CRA-032765	739
#4	Acalabrutinib OR Calquence OR ACP-196	169
#5	Zanubrutinib OR Brukinsa OR BGB-3111	91
#6	Tirabrutinib OR Velexbru OR ONO-4059 OR GS-4059	32
#7	Orelabrutinib	8
#8	Pirtobrutinib OR LOXO-305	33

#9	Nemtabrutinib OR ARQ 531 OR MK-1026	2
#10	#2 OR #3 OR #4 OR #5 OR #6 OR #7 OR #8 OR #9	1,202
#11	randomized controlled trial	970,621
#12	#1 AND #10 AND #11	448
	Total	834

Table S2. Summary of subgroup analyses of progression-free survival.

Subgroup analysis	Hazard ratio	95% Confident interval	I^2 (%)	P value
Age <65 years	0.23	0.15-0.34	0	0.92
Age ≥65 years	0.26	0.20-0.34	30	0.24
Male	0.30	0.23-0.39	6	0.34
Female	0.27	0.17-0.42	56	0.11
Raji stage 0-II or Binet stage A or B	0.26	0.15-0.45	75	0.02
Rai stage III/IV or Binet stage C	0.31	0.24-0.42	0	0.46
Bulky disease <5 cm	0.31	0.24-0.42	0	0.43
Bulky disease ≥5 cm	0.25	0.14-0.45	76	0.02
IGHV unmutated	0.22	0.14-0.35	79	< 0.01
IGHV mutated	0.61	0.42-0.90	0	0.45
Presence of del(17p)	0.15	0.08-0.26	0	0.71
Chromosome 11q deletion	0.18	0.10-0.30	31	0.24

Abbreviations: IGHV, unmutated immunoglobulin heavy chain variable; TP53, tumor protein 53.

Table S3. Summary of adverse events associated with infusion-related reactions.

Infusion-related reaction	BTK inhibitor monotherapy			Combination therapy		
	Any grade	≥Grade 3	Total	Any grade	≥Grade 3	Total
Woyach JA *et al*. 2018	NR	0	180	NR	11	176
Ghia P *et al*. 2020	1	0	154	17	3	153
Sharman JP *et al*. 2020	0	0	179	68	10	169
Tam CS *et al*. 2022	1	0	240	43	6	227

Abbreviations: BTK, Bruton tyrosine kinase; NR, not reported.

Table S4. Summary of adverse events associated with secondary primary malignancies.

Secondary primary malignancies	BTK inhibitor		Combination therapy		Risk ratio	95% Confident interval	I^2 (%)	P value
	Events	Total	Event	Total				
SPM any grade	83	573	34	549	2.49	0.64-9.70	62	0.07
**SPM of grade 3 or higher**	**45**	**753**	**20**	**725**	**2.09**	**1.01-4.36**	**0**	**0.53**
SPM excluding non-melanoma skin any grade	34	573	14	549	2.24	0.55-9.06	4	0.35
SPM excluding non-melanoma skin grade ≥3	28	573	10	549	2.58	0.86-7.77	0	0.62

Abbreviations: BTK, Bruton tyrosine kinase; SPM, secondary primary malignancies; NR, not reported.

cancers

MDPI

Table S5. PRISMA 2020 checklist.

Section and Topic	Item #	Checklist item	Location where item is reported
**TITLE**			
Title	1	Identify the report as a systematic review.	Page 1
**ABSTRACT**			
Abstract	2	See the PRISMA 2020 for Abstracts checklist.	Page 1-2
**INTRODUCTION**			
Rationale	3	Describe the rationale for the review in the context of existing knowledge.	Page 3
Objectives	4	Provide an explicit statement of the objective(s) or question(s) the review addresses.	Page 3-4
**METHODS**			
Eligibility criteria	5	Specify the inclusion and exclusion criteria for the review and how studies were grouped for the syntheses.	Page 4
Information sources	6	Specify all databases, registers, websites, organisations, reference lists and other sources searched or consulted to identify studies. Specify the date when each source was last searched or consulted.	Page 4
Search strategy	7	Present the full search strategies for all databases, registers and websites, including any filters and limits used.	Table S1
Selection process	8	Specify the methods used to decide whether a study met the inclusion criteria of the review, including how many reviewers screened each record and each report retrieved, whether they worked independently, and if applicable, details of automation tools used in the process.	Page 4
Data collection process	9	Specify the methods used to collect data from reports, including how many reviewers collected data from each report, whether they worked independently, any processes for obtaining or confirming data from study investigators, and if applicable, details of automation tools used in the process.	Page 4-5
Data items	10a	List and define all outcomes for which data were sought. Specify whether all results that were compatible with each outcome domain in each study were sought (e.g. for all measures, time points, analyses), and if not, the methods used to decide which results to collect.	Page 5
	10b	List and define all other variables for which data were sought (e.g. participant and intervention characteristics, funding sources). Describe any assumptions made about any missing or unclear information.	Page 4-5

Section and Topic	Item #	Checklist item	Location where item is reported
Study risk of bias assessment	11	Specify the methods used to assess risk of bias in the included studies, including details of the tool(s) used, how many reviewers assessed each study and whether they worked independently, and if applicable, details of automation tools used in the process.	Page 5
Effect measures	12	Specify for each outcome the effect measure(s) (e.g. risk ratio, mean difference) used in the synthesis or presentation of results.	Page 4-5
Synthesis methods	13a	Describe the processes used to decide which studies were eligible for each synthesis (e.g. tabulating the study intervention characteristics and comparing against the planned groups for each synthesis (item #5)).	Page 4-5
	13b	Describe any methods required to prepare the data for presentation or synthesis, such as handling of missing summary statistics, or data conversions.	Page 4-5
	13c	Describe any methods used to tabulate or visually display results of individual studies and syntheses.	Page 4-5
	13d	Describe any methods used to synthesize results and provide a rationale for the choice(s). If meta-analysis was performed, describe the model(s), method(s) to identify the presence and extent of statistical heterogeneity, and software package(s) used.	Page 4-5
	13e	Describe any methods used to explore possible causes of heterogeneity among study results (e.g. subgroup analysis, meta-regression).	Page 4-5
	13f	Describe any sensitivity analyses conducted to assess robustness of the synthesized results.	Page 4-5
Reporting bias assessment	14	Describe any methods used to assess risk of bias due to missing results in a synthesis (arising from reporting biases).	Page 4-5
Certainty assessment	15	Describe any methods used to assess certainty (or confidence) in the body of evidence for an outcome.	Page 4-5
**RESULTS**			
Study selection	16a	Describe the results of the search and selection process, from the number of records identified in the search to the number of studies included in the review, ideally using a flow diagram.	Page 5-6
	16b	Cite studies that might appear to meet the inclusion criteria, but which were excluded, and explain why they were excluded.	Page 5-6
Study characteristics	17	Cite each included study and present its characteristics.	Page 5-7
Risk of bias in studies	18	Present assessments of risk of bias for each included study.	Page 11

Section and Topic	Item #	Checklist item	Location where item is reported
Results of individual studies	19	For all outcomes, present, for each study: (a) summary statistics for each group (where appropriate) and (b) an effect estimate and its precision (e.g. confidence/credible interval), ideally using structured tables or plots.	Page 6-8
Results of syntheses	20a	For each synthesis, briefly summarise the characteristics and risk of bias among contributing studies.	Page 7-13
	20b	Present results of all statistical syntheses conducted. If meta-analysis was done, present for each the summary estimate and its precision (e.g. confidence/credible interval) and measures of statistical heterogeneity. If comparing groups, describe the direction of the effect.	Page 9-14
	20c	Present results of all investigations of possible causes of heterogeneity among study results.	Page 9-14
	20d	Present results of all sensitivity analyses conducted to assess the robustness of the synthesized results.	Page 9-14
Reporting biases	21	Present assessments of risk of bias due to missing results (arising from reporting biases) for each synthesis assessed.	Page 11-13
Certainty of evidence	22	Present assessments of certainty (or confidence) in the body of evidence for each outcome assessed.	Page 9-14
**DISCUSSION**			
Discussion	23a	Provide a general interpretation of the results in the context of other evidence.	Page 14-15
	23b	Discuss any limitations of the evidence included in the review.	Page 16
	23c	Discuss any limitations of the review processes used.	Page 16
	23d	Discuss implications of the results for practice, policy, and future research.	Page 16-17
**OTHER INFORMATION**			
Registration and protocol	24a	Provide registration information for the review, including register name and registration number, or state that the review was not registered.	Page 4
	24b	Indicate where the review protocol can be accessed, or state that a protocol was not prepared.	Page 4
	24c	Describe and explain any amendments to information provided at registration or in the protocol.	Page 4
Support	25	Describe sources of financial or non-financial support for the review, and the role of the funders or sponsors in the review.	Page 18
Competing interests	26	Declare any competing interests of review authors.	Page 18
Availability of data, code and	27	Report which of the following are publicly available and where they can be found: template data collection forms; data extracted from included studies; data used for all analyses; analytic code; any other materials used in the review.	Page 18

Section and Topic	Item #	Checklist item	Location where item is reported
other materials			

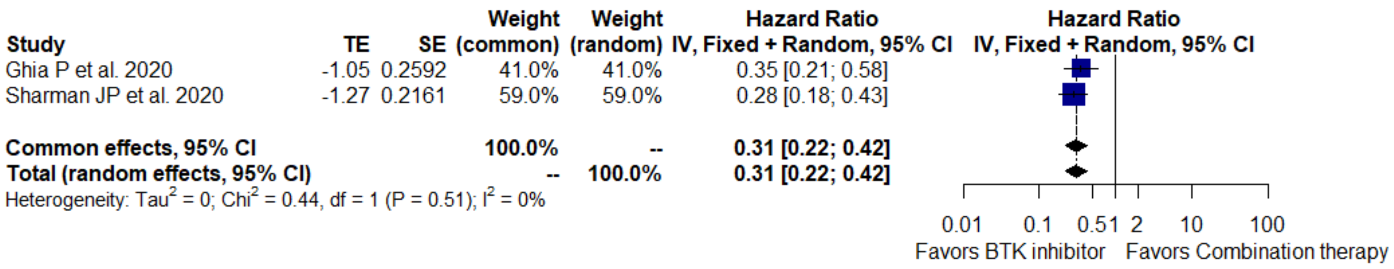

Figure S1. Forest plot for time to next treatment [31,43].

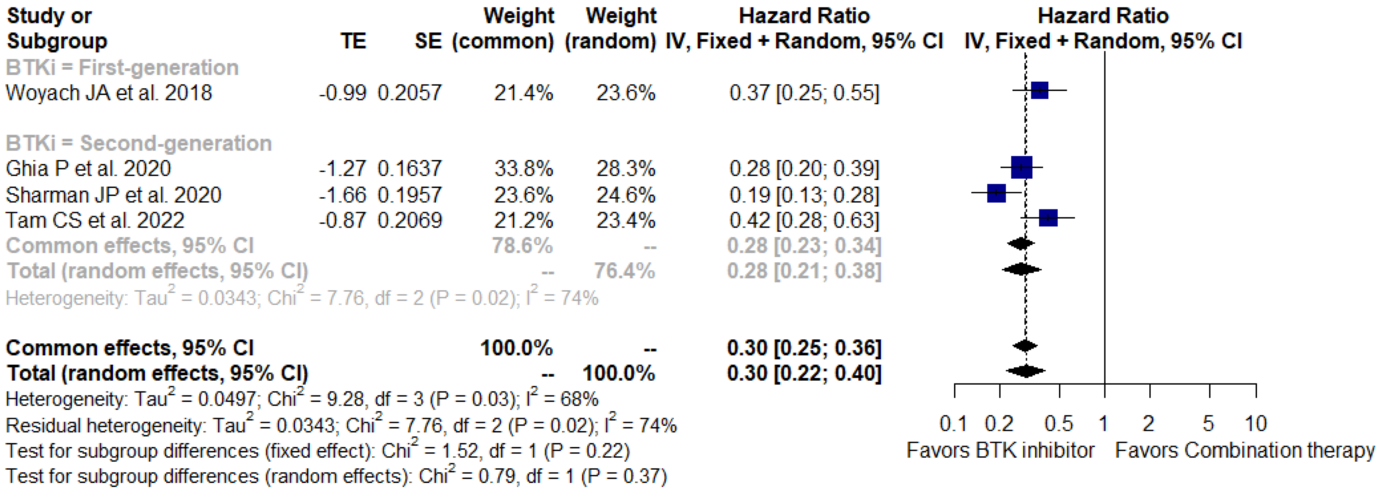

Figure S2. Forest plot for progression-free survival (subgroup different generation BTK inhibitors) [26,31,43,46].

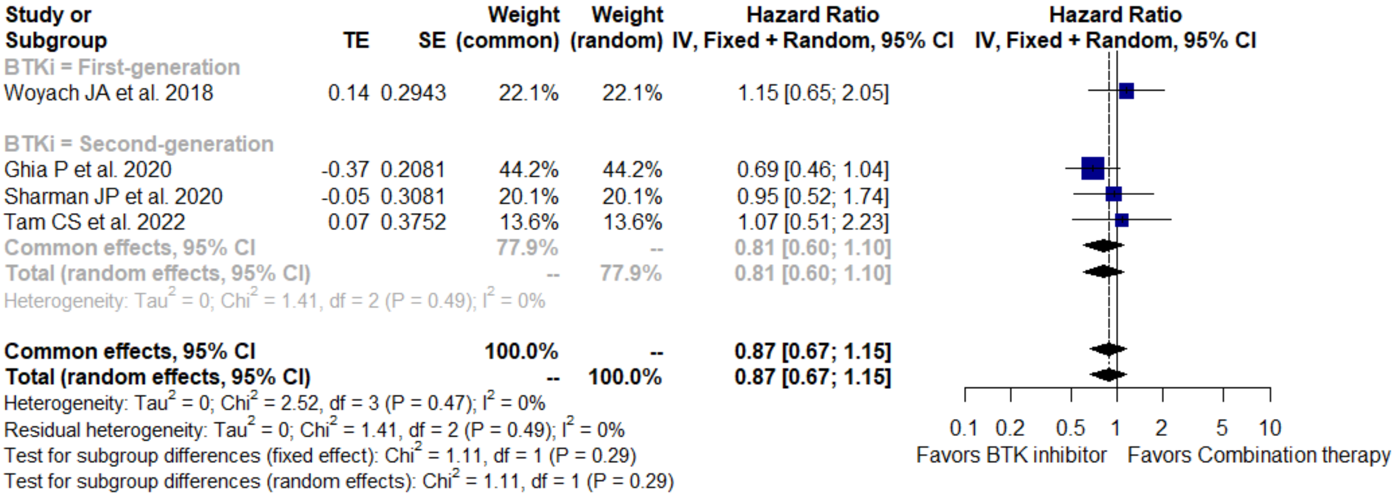

Figure S3. Forest plot for overall survival (subgroup different generation BTK inhibitors) [26,31,43,46].

(A)

Study or Subgroup	Experimental Events	Experimental Total	Control Events	Control Total	Weight (common)	Weight (random)	Risk Ratio MH, Fixed + Random, 95% CI
BTKi = First-generation							
Woyach JA et al. 2018	169	182	148	183	23.3%	20.4%	1.15 [1.06; 1.25]
BTKi = Second-generation							
Ghia P et al. 2020	143	155	136	155	21.4%	24.2%	1.05 [0.98; 1.13]
Sharman JP et al. 2020	161	179	146	177	23.1%	19.1%	1.09 [1.00; 1.19]
Tam CS et al. 2022	228	241	203	238	32.2%	36.3%	1.11 [1.04; 1.18]
Common effects, 95% CI		575		570	76.7%	--	1.09 [1.04; 1.13]
Total (random effects, 95% CI)					--	79.6%	1.09 [1.01; 1.17]
Heterogeneity: $Tau^2 = 0$; $Chi^2 = 1.2$, df = 2 (P = 0.55); $I^2 = 0\%$							
**Common effects, 95% CI**		**757**		**753**	**100.0%**	**--**	**1.10 [1.06; 1.14]**
**Total (random effects, 95% CI)**					**--**	**100.0%**	**1.10 [1.04; 1.16]**

Heterogeneity: $Tau^2 = 0$; $Chi^2 = 2.59$, df = 3 (P = 0.46); $I^2 = 0\%$
Residual heterogeneity: $Tau^2 = 0$; $Chi^2 = 1.20$, df = 2 (P = 0.55); $I^2 = 0\%$
Test for subgroup differences (fixed effect): $Chi^2 = 1.37$, df = 1 (P = 0.24)
Test for subgroup differences (random effects): $Chi^2 = 1.52$, df = 1 (P = 0.22)

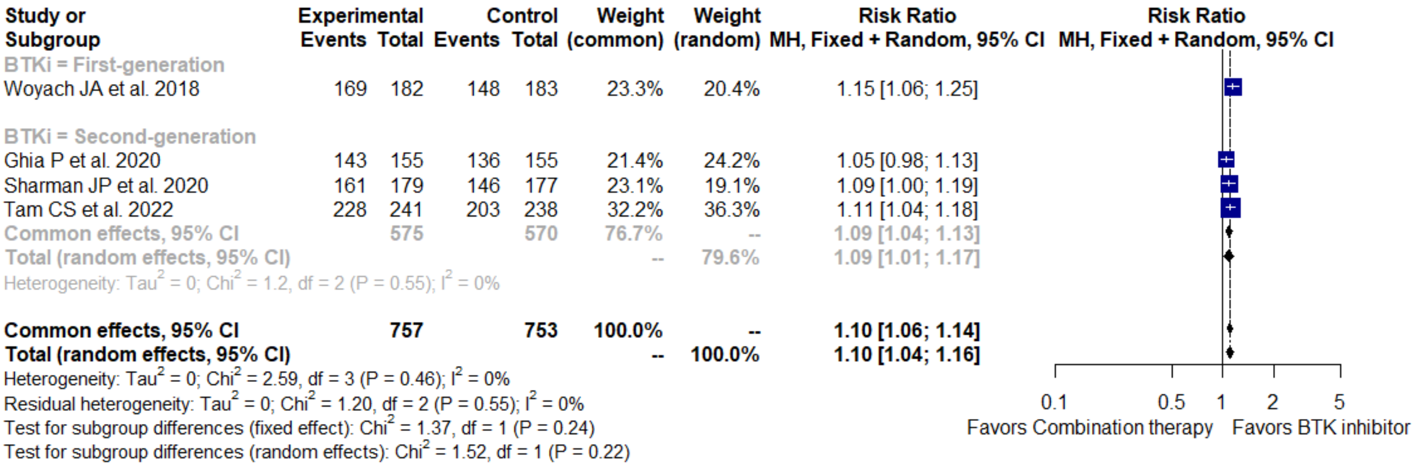

(B)

Study or Subgroup	Experimental Events	Experimental Total	Control Events	Control Total	Weight (common)	Weight (random)	Risk Ratio MH, Fixed + Random, 95% CI
BTKi = First-generation							
Woyach JA et al. 2018	12	182	48	183	41.2%	26.1%	0.25 [0.14; 0.46]
BTKi = Second-generation							
Ghia P et al. 2020	9	155	9	155	7.7%	20.0%	1.00 [0.41; 2.45]
Sharman JP et al. 2020	20	179	23	177	19.9%	26.9%	0.86 [0.49; 1.51]
Tam CS et al. 2022	16	241	36	238	31.2%	26.9%	0.44 [0.25; 0.77]
Common effects, 95% CI		575		570	58.8%	--	0.66 [0.46; 0.94]
Total (random effects, 95% CI)					--	73.9%	0.68 [0.23; 2.04]
Heterogeneity: $Tau^2 = 0.0833$; $Chi^2 = 3.71$, df = 2 (P = 0.16); $I^2 = 46\%$							
**Common effects, 95% CI**		**757**		**753**	**100.0%**	**--**	**0.49 [0.36; 0.66]**
**Total (random effects, 95% CI)**					**--**	**100.0%**	**0.54 [0.20; 1.46]**

Heterogeneity: $Tau^2 = 0.2876$; $Chi^2 = 11.13$, df = 3 (P = 0.01); $I^2 = 73\%$
Residual heterogeneity: $Tau^2 = 0.0833$; $Chi^2 = 3.71$, df = 2 (P = 0.16); $I^2 = 46\%$
Test for subgroup differences (fixed effect): $Chi^2 = 7.25$, df = 1 (P < 0.01)
Test for subgroup differences (random effects): $Chi^2 = 4.15$, df = 1 (P = 0.04)

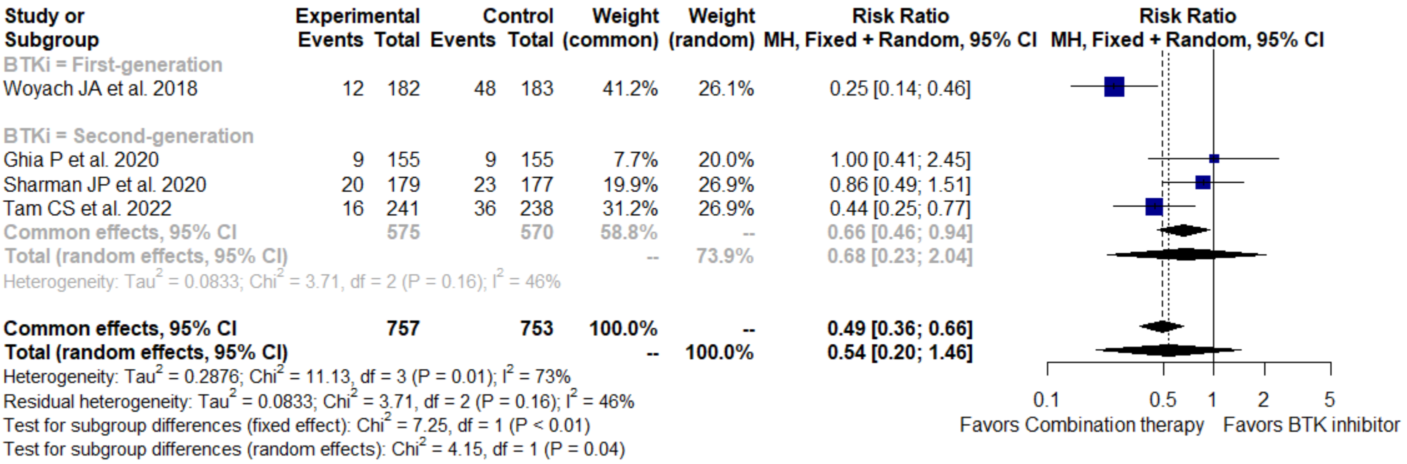

Figure S4. Pooled risk ratios for overall response (A) and complete response (B) (subgroup different generation BTK inhibitors) [26,31,43,46].

Study or Subgroup	Experimental Events	Experimental Total	Control Events	Control Total	Weight (common)	Weight (random)	Risk Ratio MH, Fixed + Random, 95% CI
BTKi = First-generation							
Woyach JA et al. 2018	133	180	111	176	20.6%	25.2%	1.17 [1.02; 1.35]
BTKi = Second-generation							
Ghia P et al. 2020	104	154	125	153	23.0%	25.4%	0.83 [0.72; 0.94]
Sharman JP et al. 2020	89	179	118	169	22.3%	24.1%	0.71 [0.60; 0.85]
Tam CS et al. 2022	126	240	181	227	34.1%	25.3%	0.66 [0.57; 0.76]
Common effects, 95% CI		573		549	79.4%	--	0.72 [0.66; 0.79]
Total (random effects, 95% CI)					--	74.8%	0.73 [0.54; 0.98]
Heterogeneity: $Tau^2 = 0.0086$; $Chi^2 = 5.6$, df = 2 (P = 0.06); $I^2 = 64\%$							
**Common effects, 95% CI**		**753**		**725**	**100.0%**	**--**	**0.81 [0.76; 0.88]**
**Total (random effects, 95% CI)**					**--**	**100.0%**	**0.82 [0.55; 1.23]**

Heterogeneity: $Tau^2 = 0.0597$; $Chi^2 = 36.28$, df = 3 (P < 0.01); $I^2 = 92\%$
Residual heterogeneity: $Tau^2 = 0.0086$; $Chi^2 = 5.60$, df = 2 (P = 0.06); $I^2 = 64\%$
Test for subgroup differences (fixed effect): $Chi^2 = 32.55$, df = 1 (P < 0.01)
Test for subgroup differences (random effects): $Chi^2 = 11.92$, df = 1 (P < 0.01)

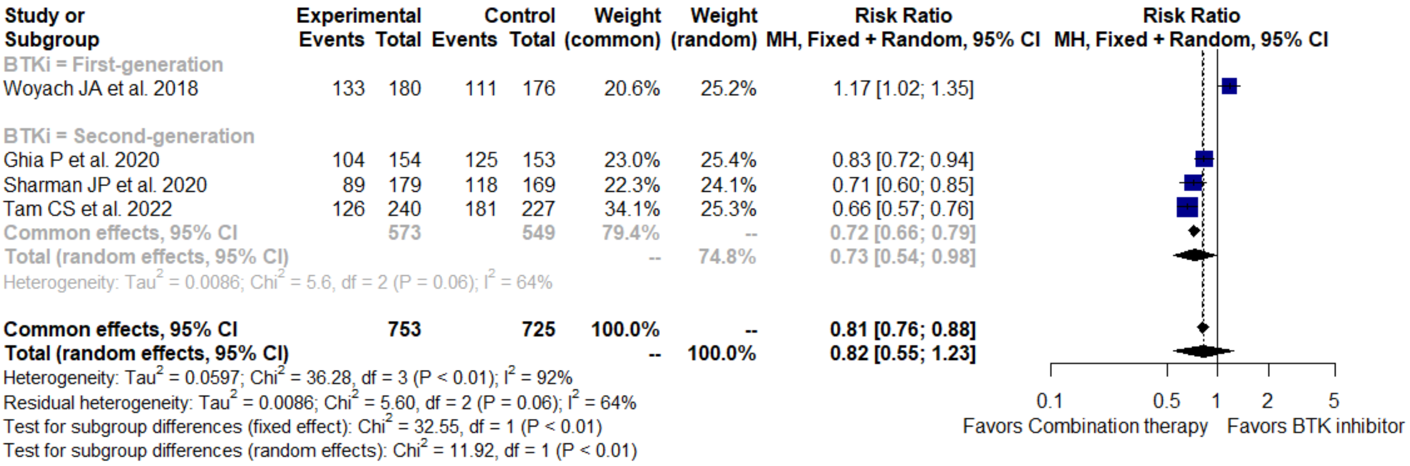

Figure S5. Pooled risk ratios for grade ≥3 adverse events [26,31,43,46].
